# Electron Compton scattering and the measurement of electron momentum distributions in solids

**DOI:** 10.1111/jmi.12854

**Published:** 2020-01-06

**Authors:** A. TALMANTAITE, M.R.C. HUNT, B.G. MENDIS

**Affiliations:** ^1^ Centre for Materials Physics Durham University Durham United Kingdom

**Keywords:** Compton scattering, density distribution, electron energy‐loss spectroscopy, electron momentum, impulse approximation

## Abstract

Electron Compton scattering is a technique that gives information on the electron momentum density of states and is used to characterize the ground state electronic structure in solids. Extracting the momentum density of states requires us to assume the so‐called ‘impulse approximation’, which is valid for large energy losses. Here, the robustness of the impulse approximation in the low energy transfer regime is tested and confirmed on amorphous carbon films. Compared to traditional Compton measurements, this provides additional benefits of more efficient data collection and a simplified way to probe valence electrons, which govern solid state bonding. However, a potential complication is the increased background from the plasmon signal. To overcome this, a novel plasmon background subtraction routine is proposed for samples that are resistant to beam damage.

**Lay Description:**

Properties of solids depend on their electronic structure which can be studied using electron Compton scattering technique. Here, an electron beam is used to penetrate a very thin sample. During the interaction between the electrons in the beam and electrons in the sample, the former transfer a part of their energy to the latter, resulting in a measurable energy loss of the transmitted beam. The amount of the energy transfer depends on the angle of incidence between the beam and the sample. Typically, the experiments are carried out using high tilt angles and high energy transfer; however, in this work, we show that even smaller angles of incidence are suitable, which improve the signal quality and ease data processing procedures.

## Introduction

The physical properties of solids are determined exclusively by the electronic density of states and, therefore, development of techniques that measure the ground state electronic properties at high spatial resolution is essential (Martin & Martin, 2004). Electron Compton scattering can characterize electronic structure of solids by providing information on the electronic density of states resolved as a function of electron momentum (Cooper, 1985). This also enables experimental verification of band structure diagrams predicted by density functional theory (DFT) simulations (Martin & Martin, 2004). Electron Compton scattering offers three major advantages over the traditional photon‐based experiments: orders of magnitude higher inelastic scattering cross‐section, a sample size threshold as small as a few nanometres in area, and compatibility with simultaneous electron diffraction and energy dispersive X‐ray measurements (Feng *et al*., 2013). Currently, Compton scattering experiments are carried out in a high energy transfer regime (i.e. significantly above the binding energy of the recoil electron) in order to satisfy the impulse approximation (see Section ‘Impulse approximation theory’). However, this results in a low electron count rate, while the Compton profiles thus obtained include contributions from both valence and core electrons, where the latter has to be calculated using DFT methods and subtracted (Feng *et al*., 2013, 2015, 2016).

In this work, Compton scattering experiments on amorphous carbon films are carried out using a transmission electron microscope (TEM) equipped with an electron energy loss spectrometer. Particular emphasis is placed on determining the validity of the impulse approximation in the `low energy' transfer regime at small electron scattering angles. In this regime, the Compton peak is at a lower energy loss than the carbon K‐edge, so that only valence electrons are Compton scattered. We also address background subtraction techniques and compare Compton profiles at both low and high energy transfers.

## Impulse approximation theory

Interpretation of the Compton spectrum in terms of the momentum distribution of target electrons relies on the validity of the impulse approximation, which assumes that relaxation of background electrons and nuclei in the target is negligible during the collision (Williams *et al*., 1984; Cooper, 1985). Under this condition, any changes in the potential energy can be ignored. In TEM, where highly energetic electron beams are accelerated to a substantial fraction of the speed of light, relativistic effects must be considered, giving the following energy and momentum conservation relations:

(1) $$E_{1}+\;E_{i}=E_{2}\;+E_{f},$$

(2) $$p_{1}+\;p_{i}=p_{2}\;+p_{f},$$

(3) $$\;E^{2}={\left(T+m_{0}c^{2}\right)}^{2}\;={\left(pc\right)}^{2}\;+{\left(m_{0}c^{2}\right)}^{2},$$

where *E*_1_, **p**_1_ and *E*_2_, **p**_2_ are the energies and momenta of the primary and scattered electron; *E*_i_, **p**_i_ and *E*_f_, **p**_f_ are the initial and final energies and momenta of the target electron; *T* is the kinetic energy of an electron of rest mass *m*_0_ and *c* is the speed of light. Equations (1) to (3) can be used to obtain the relation linking the energy, *ΔT*, and momentum, *Δ* **p**, transferred by the primary electron:

(4) $$\Delta T\left(m_{0}c^{2}\right)\;=\frac{1}{2}c^{2}{\left|\Delta p\right|}^{2}-\;\frac{1}{2}\left(\Delta T^{2}\right)-c^{2}p_{i}\cdot \Delta p-\;T_{i}\Delta T.\;$$

Further, assuming that$\;|p_{1}\left|≅\right|p_{2}|$, the magnitude of the momentum transfer is given by $\left|\Delta p|=\;2\right|p_{1}|\sin\frac{\varphi }{2}$. Substituting into Eq. (4) and setting both the initial kinetic energy of the target electron, *T_i_*, and initial momentum, **p**_i_, to zero gives the energy transfer at the peak of the Compton profile, *ΔT*_p_, as a function of the scattering angle, φ:

(5) $$\Delta \;T_{p}=\;2\mathrm{si}n^{2}\frac{\varphi }{2}\left(2T_{1}+\frac{T_{1}^{2}}{m_{0}c^{2}}\right).$$

In deriving Eq. (5), it is assumed that $\Delta T\ll m_{0}c^{2}$, so that the $\frac{1}{2}\Delta T^{2}$ term in Eq. (4) can be neglected.

In order to express the energy transfer in terms of the momentum, *p*_z_, of a target electron projected along the scattering vector, one can substitute $\Delta T\;=\;\Delta T_{p}+\delta T$ into Eq. (4), where the term $T_{i}\Delta T$ can be neglected since $T_{i}\ll m_{0}c^{2}$. Considering that$\;\Delta T_{p}\;\left(m_{0}c^{2}\right)=\frac{1}{2}\;c^{2}{\left|\Delta p\right|}^{2}-\frac{1}{2}\left(\Delta T^{2}\right)$, we obtain:

(6) $$\;p_{z}=\frac{-\delta Tm_{0}}{\left|\Delta p\right|}\;=\;-\delta T\sqrt{\frac{m_{0}}{2\Delta T_{p}}}.$$

## Experimental methods

The Compton spectra of amorphous holey carbon films were obtained using a JEOL 2100F TEM equipped with a Gatan Tridiem imaging filter. The specimens were probed with a 197 keV electron beam in a centred dark field mode at angles of incidence ranging between 23 and 46 mrad. Compton spectra were acquired at energy dispersions of 0.3–0.5 eV/channel. The specimen was approximately 10 nm thick as measured using electron energy‐loss spectroscopy (EELS).

## Results and discussion

### Impulse approximation

**Figure 1:**
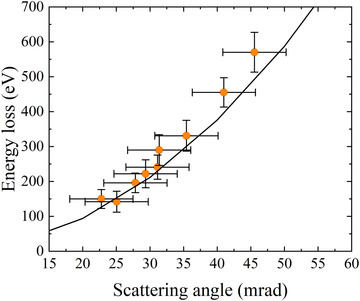
The measured values of energy loss at the peaks of Compton spectra across a range of beam tilt angles (points) compared with the predictions of the impulse approximation (solid line).

The validity of the impulse approximation was investigated by comparing the measured values of electron energy loss at the peaks of Compton spectra with the values predicted by Eq. (5). Generally, the approximation is expected to break down in the low energy transfer regime (Cooper, 1985). However, Fig. 1 shows that the observed scattering behaviour is described by the impulse approximation even at low scattering angles, between 20 and 30 mrad, where the energy transfer is below the carbon K‐shell binding energy. Core hole effects can introduce peak shifts of up to a few electronvolts in EELS core loss edges (Feng *et al*., 2015, 2016). Assuming a 1 eV peak shift due to breakdown of the impulse approximation gives an uncertainty of only approximately 0.01 atomic units in *p*_z_ (Eq. (6)) for the smallest scattering angle in Fig. 1. Therefore, we expect Compton profiles in the low energy transfer regime to still provide accurate measurements. Following appropriate plasmon subtraction methods discussed below, these spectra can be transformed into momentum density profiles (Eq. (6)) containing the contributions only from the valence electrons.

### Plasmon background subtraction and *J*(*p_z_*) profiles

**Figure 2:**
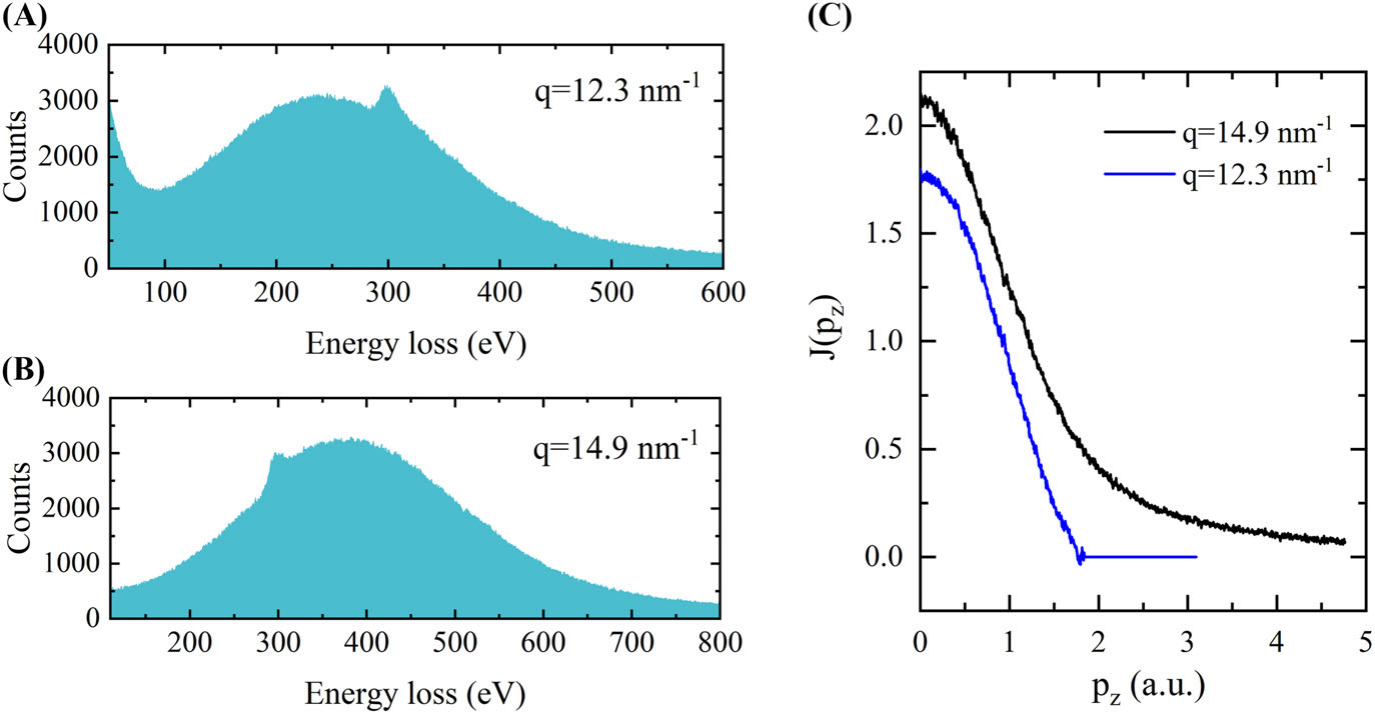
EELS spectra of amorphous carbon films in the low energy transfer, (A), and high energy transfer regimes, (B), and the corresponding Compton profiles in (C).

In order to obtain projected momentum density profiles, *J(p_z_)*, of amorphous carbon films we used a power law defined plasmon background subtraction. Figure 2C shows the resulting Compton profiles, *J(p_z_)*, measured in the high energy transfer (black) and low energy transfer (blue) regimes. The decay length of the Compton profile obtained in the hard collision regime is longer due to the contribution from the core electrons. Furthermore, Compton profiles are subject to a normalisation rule, which equates the integral of the profile to the number of electrons participating in scattering (Cooper, 1985). Here, integration of the Compton profile at the low energy transfer yielded 1.8 electrons, which is in quantitative agreement with the expected 2 valence electrons for carbon per half of the Compton profile. The *J(p_z_)* for valence electrons is also similar to that reported for graphite (Feng *et al*., 2013).

**Figure 3:**
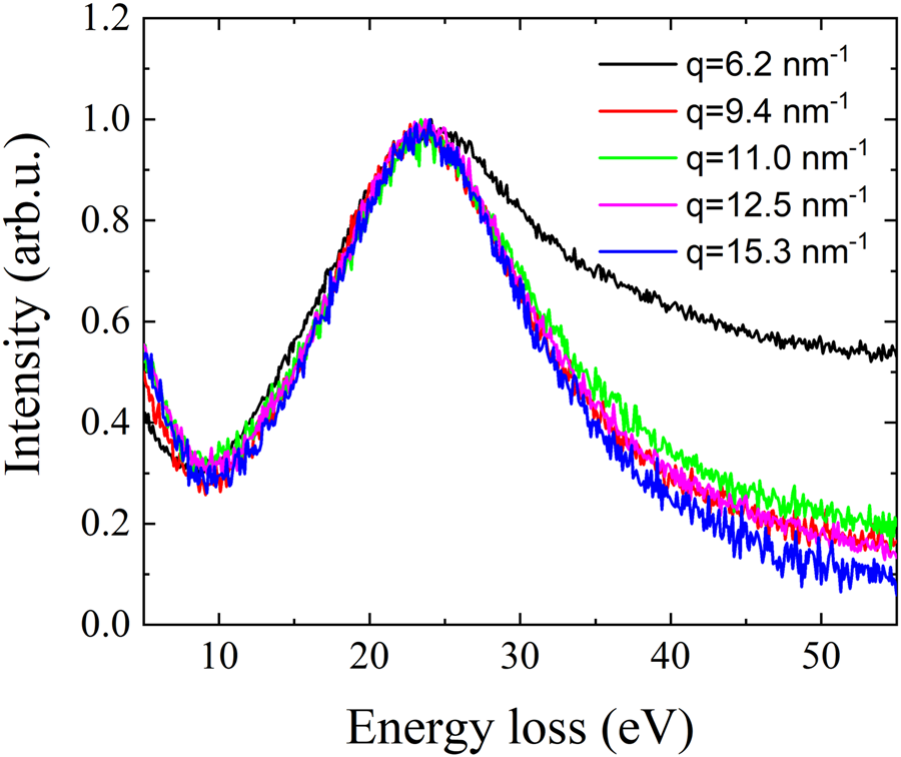
Plasmon peaks measured at a range of different energy and momentum transfer values. The increase in intensity at the high‐energy tale at momentum transfer *q* = 6.2 nm^−1^ is an artefact from a Compton peak located at a close proximity to the plasmon peak. Furthermore, the spectra for *q* = 9.4 nm^−1^ and *q* = 12.5 nm^−1^ are strongly overlapping.

The background subtraction using the power law is generally considered to be a fairly crude way to remove the plasmon contribution, we further examined potential plasmon background subtraction techniques by looking at the behaviour of plasmon spectra over a number of different scattering angles. The cross‐section for plasmon excitations is defined by a Lorentzian distribution, the peak of which is expected to be shifted to higher energy loss with an increasing scattering angle (Egerton, 2011). However, as shown in Fig. 3, a near constant plasmon shape and peak position is observed, which suggests that these spectra originate mainly from double scattering rather than single inelastic scattering events. In other words, the (tilted) primary electron beam is elastically scattered towards the optic axis and also undergoes low angle plasmon inelastic scattering. The single inelastic scattering contribution is expected to be negligible due to the narrow Lorentzian distribution for plasmon excitation. In this way, the plasmons measured at the relatively large beam tilt angles used for Compton scattering are essentially identical to plasmons measured at a zero tilt angle.

**Figure 4:**
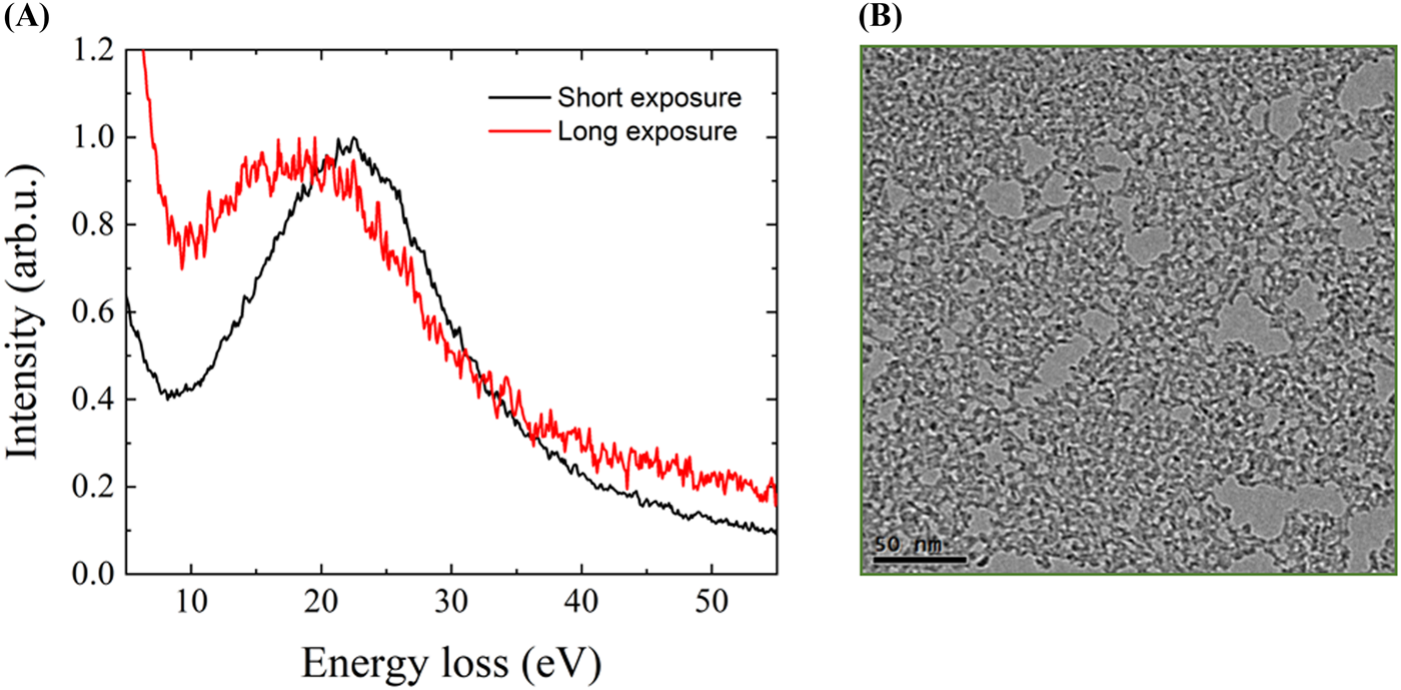
Sample damage caused by long exposure to electron beam causes widening and shifting of the plasmon peak (peak intensity is normalized) (A). (B) Shows the corresponding highly damaged sample area.

In principle, the plasmon background can be removed by subtracting a suitably normalized zero‐tilt EELS spectrum from the tilted spectrum containing the Compton profile. However, this method did not produce satisfactory results for our measurements. Close examination of the data revealed that this is due to small amount of beam damage of the amorphous carbon film during prolonged acquisition of the Compton spectrum. This is illustrated in Fig. 4A, which compares plasmon spectra obtained at short and long exposure times. The latter was made deliberately longer than the typical acquisition time for the Compton profile in order to clearly visualize the changes occurring due to beam damage. The plasmon peak at a longer acquisition time is broader and shifted to lower energy loss. The amorphous carbon film after long exposure (Fig. 4B) shows sputter damage and formation of voids. The plasmon peak energy is predicted to vary monotonically with valence electron density, while the width of the peak is dependent on the plasmon lifetime (Egerton, 2011). Vacancies produced by sputter damage will reduce the valence electron density and decrease the plasmon lifetime. This is consistent with the observations in Fig. 4A; similar results have been reported for ion irradiation of carbon‐based materials (Brzhezinskaya *et al*., 2004, 2005). The subtle effect of beam damage precludes accurate plasmon background subtraction in our experiments. Therefore, the described background subtraction technique can only be used on more robust materials or with beam energies below the knock‐on threshold.

## Conclusions

We have derived relativistic equations describing electron Compton scattering within the conditions of the impulse approximation and confirmed that the approximation is still appropriate in the low energy transfer regime. Here, only valence electrons participate in scattering and provide momentum density profiles for which core electron background subtraction using DFT is no longer required. The higher count rate of the EELS spectrum is a further advantage of low energy transfers. We have also outlined a method for plasmon background subtraction for samples resistant to electron beam damage and demonstrated that even a crude plasmon background subtraction using a power law provides a reasonable match with the expected results.
